# A Comparison of Monoglyceride Production from Microalgaelipids and Rapeseed Oil Catalyzed by Metal Oxides

**DOI:** 10.1002/cssc.202400953

**Published:** 2024-08-14

**Authors:** Gabriela F. Ferreira, Luisa F. Ríos Pinto, Rubens Maciel Filho, Leonardo V. Fregolente, James S. Hayward, Jonathan K. Bartley

**Affiliations:** ^1^ Department of Processes and Products Development School of Chemical Engineering University of Campinas 500 Albert Einstein Av Campinas 13085 045 São Paulo Brazil; ^2^ Cardiff Catalysis Institute School of Chemistry Cardiff University Park Place Cardiff CF10 3AT Wales United Kingdom

**Keywords:** microalgae, lipid, glycerolysis, heterogeneous catalysis, polyunsaturated fatty acids

## Abstract

This manuscript reports for the first time a heterogenous catalytic route to monoglycerides (MAGs) from microalgal oil. Microalgae is an important biomass source with high‐value applications, such as food ingredients with essential fatty acids. To date, the glycerolysis of microalgae has only been investigated for a microbial oil (*Schizochytrium* sp.) using enzyme catalysis. However, the use of enzymes on a large scale is currently economically impeditive and requires highly selective lipases. In this study, metal oxides were screened and the reaction conditions optimized for rapeseed oil. The optimized conditions were then used to investigate the production of MAGs from *Scenedesmus* sp. microalga. The most promising catalyst was found to be MgO/KOH, which gave a 44 % yield. Comparing two reaction systems (low temperature 70 °C/atmospheric pressure and high temperature at 200 °C/20 bar), it was found that the latter has a superior performance. Due to the stability of the product in air, the presence of an inert atmosphere is essential to achieve high yields.

## Graphical abstract

Microalgae is an underutilised source of food with high nutritional value, such as omega 3, −6, and −9. Glycerolysis of microalgal oil is a simple route to obtain emulsifiers rich in these essential fatty acids and this study reports the first use of heterogeneous catalysts for this application.

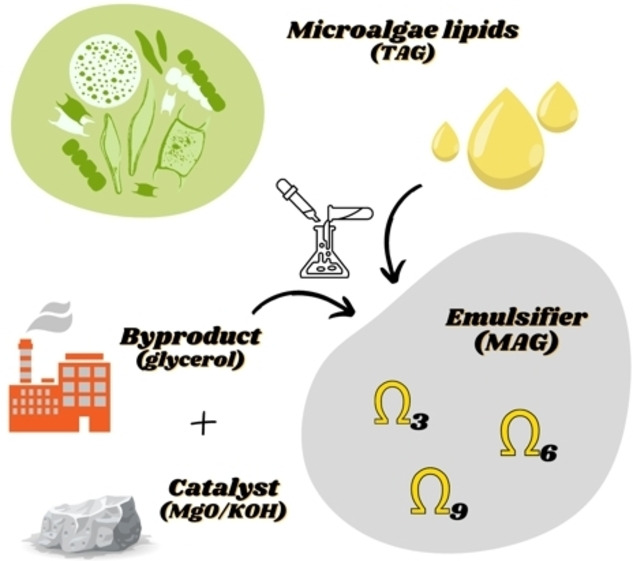

## Introduction

Microalga lipids have been extensively studied for biodiesel production employing different catalytic routes. Homogeneous catalysts are the most used, either for the esterification of free fatty acids (FFAs) using an acid catalyst or transesterification of triglycerides (TAGs) using a base catalyst. The latter is usually preceded by neutralization due to high free fatty acid content in microalgae. Alternative studies have investigated heterogeneous catalysts (metal oxides, molecular sieves, and other solids), or enzymes (mostly selective lipases) for the conversion of FFAs and TAGs.^[1]^ However, the high capital expenditure and operational costs of microalgae large‐scale cultivation lead to a process that is not competitive for biodiesel production, according to economic assessments.[^2^, ^3^] Consequently, it is imperative to study how to reduce costs associated with upstream and downstream processing as well as higher value applications to encourage market interest. Some microalgae species are known to have valuable components in their lipid fraction, which are of interest to pharmaceutical, nutraceutical, and cosmetic industries.^[4]^ Microalgal biomass can be a source of potential nutraceutical compounds and food supplements for human health such as essential amino and/or fatty acids, pigments, and vitamins.^[5]^ Furthermore, a lipid fraction rich in omega‐3, ‐6, and ‐9 fatty acids can be found in many species, from both seawater and freshwater, which could integrate the use of wastewater as a source of nutrients to reduce cultivation costs.^[6]^ Other value‐added bioproducts can also be obtained such as organic acids^[7]^ or less conventional products such as a polyester produced from crude algae oil.^[8]^

A further way to reduce the cost of microalgae valorisation is process integration with existing industries. Glycerol, the transesterification reaction main byproduct, has several value‐added applications^[9]^ and could be used as a carbon source during cultivation of microalgae or as a starting material to obtain other bioproducts. Glycerol has been reported to improve simultaneously the microalgae growth and TAGs accumulation,^[10]^ being used as both a carbon source and energy source that favours the production of this metabolite. Alternatively, glycerolysis, a reaction between TAGs and glycerol, produces monoglycerides (MAGs) when excess glycerol is used, which are emulsifiers with several applications in the food industry. For example, they can be applied as an emulsifier, stabiliser, or conditioning agent while having nutritional value.^[1]^ The production of mono‐ and diglycerides (DAGs) rich in omega‐3 fatty acids has already been considered from microalgal biomass, but the studies approaching glycerolysis or ethanolysis are still very limited and restricted to using lipases as catalysts and the mechanisms for the reaction pathways have been previously reviewed by Ferreira et al.^[1]^ Enzymatic routes are often chosen because they exhibit high conversion and selectivity,^[11]^ and require much lower temperatures than homogeneous and heterogeneous routes. Although these characteristics would be advantageous for microalgae lipids with high polyunsaturated fatty acid (PUFA) levels to avoid degradation, they are not economically competitive at present.

Currently, the most used catalysts for the conversion of vegetable oils into MAGs and DAGs are homogeneous bases such as KOH and NaOH that can reach up to 90%.^[12]^ However, homogeneous catalysts are associated with high temperatures (>200 °C), equipment corrosion, safety issues, soap formation, non‐reusability, and require neutralisation.^[13]^ Seeking alternatives to their use, researchers have shown that heterogeneous catalysts have great potential as they are environmentally friendly with high activity and can be used repeatedly.^[12]^

The use of solid catalysts has been widely studied for vegetable oils[^14^, ^15^, ^16^, ^17^, ^18^, ^19^, ^20^, ^21^] and represents a solution to the problems associated with homogeneous base catalysts. They have the advantage of easy separation and reuse potential and can be produced from waste material as is the case of CaO.^[22]^ The mechanism of the glycerolysis over basic metal oxide catalysts has been has been investigated using both experimental and theoretical methods. Studies on MgO have suggested that the initial step is proton extraction from the glycerol by a strongly basic O^2‐^ which are found in low coordination edge and corner sites.[^15^, ^16^] This is more favourable on the secondary hydroxyl group to give β‐glyceroxide, although dissociation at the terminal hydroxyl can also occur to give α‐glyceroxide. Following the dissociative adsorption the glyceroxide anions are then stabilised on the Mg^2+^ Lewis acid sites. These Lewis acidic sites can also activate the TAG, which adsorbs to the Mg^2+^ through the carbonyl group, polarising the carbon‐oxygen bond, making electrophilic attack of the hydroxyl more favourable on the C^δ+^ atom.^[15]^ Due to steric hindrance the reaction is more likely at the terminal position resulting in formation of α‐MAGs over β‐MAGs.^[15]^ A similar mechanism has been proposed by Ong et al. using a homogeneous/heterogenous system that used NaOH to activate the glycerol molecule and CuO to activate the TAG.^[19]^

However, most heterogeneous catalysis studies involving lipids extracted from microalgal are restricted to transesterification reactions, while enzymatic routes were tested for both biodiesel[^23^, ^24^, ^25^, ^26^] and emulsifier production.[^27^, ^28^, ^29^, ^30^, ^31^, ^32^] Although the transesterification of microalgae has been widely studied, heterogeneous glycerolysis is a promising route, yet to be explored, as a means of generating a higher process revenue.

Some studies have investigated metal oxide catalysts to obtain MAGs and DAGs from vegetable oils under mild conditions, although lower reaction rates and MAGs selectivity were achieved using solid catalysts compared to NaOH.^[14]^ Although the stoichiometry of the reaction suggests that two molecules of glycerol are required to form three molecules of MAG, excess glycerol is usually used to shift the equilibrium concentration. Corma et al. suggest that glycerol/oil ratios of between 6 and 12 give the maximum conversion and selectivity, while ratios greater than 12 can complicate ester and glycerol recovery adding to the cost of the process.^[17]^ A few other strategies have been found to improve the reaction yield using heterogeneous catalysts and inhibit thermal degradation and oxidation, such as the addition of antioxidants or carrying out the reaction under an inert atmosphere (N_2_).^[33]^ As previously stated, solid catalysts have not been tested for microalgal oil and this research topic is deficient in the literature.^[1]^ To date, microalgae glycerolysis has only been assessed as an upstream processing step of transesterification to reduce free fatty acids content,^[34]^ rather than as a route to high value products.

In the present study, we have explored different basic and acidic metal oxides as catalysts for the glycerolysis of a vegetable (rapeseed) oil and, for the first time, microalgae oil (*Scenedesmus* sp.). Synthesis methodologies were selected to optimize surface area and activity. After the initial screening, the most promising metal oxide catalyst was investigated in detail, and reaction parameters were evaluated by statistical analysis followed by a time‐on‐line study. This screening of process conditions was performed using commercial rapeseed oil, with the optimized reaction conditions then applied for the glycerolyisis of microalgal oil that had been previously extracted and purified from dry biomass. No prior studies using microalgal biomass for heterogeneous glycerolysis have been reported in the literature according to a recent review^[1]^ and subsequent literature searches Therefore, to the best of our knowledge, this is the first time that a systematic study of microalgal glycerolysis oil catalysed by metal oxides has been carried out and the reuse potential of MgO/KOH explored.

## Results and Discussion

### Glycerolysis Reaction of Rapeseed Oil

**Figure 1:**
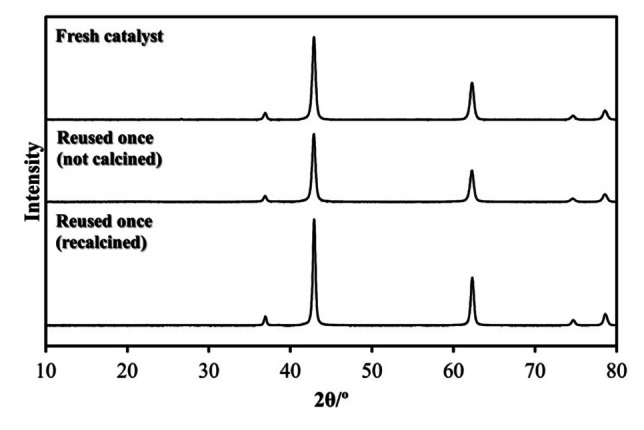
XRD of the MgO/KOH catalyst before and after use.

Initially, single metal oxide catalysts, with basic (CaO, MgO, and La_2_O_3_) or acidic (Nb_2_O_5_, SiO_2_, and WO_3_) properties were screened for the glycerolysis reaction using a stoichiometric amount of glycerol. However, strong basic or acidic sites are needed to deprotonate the glycerol in the intial step of the reaction[^15^, ^16^, ^17^] and these single oxide materials did not show any triglyceride conversion, so catalysts with higher basicity (MgO/KOH) and acidity (Nb_2_O_5_/H_3_PO_4_) were investigated. The surface areas of these materials were determined from nitrogen physisorption and found to be 40 and 4.1 m^2^ g^−1^, with pore radii of 14.99 and 17.41 Å, respectively, and can both be classified as microporous.^[35]^ Comparing the performance of the two catalysts, only the basic metal oxide exhibited a MAGs yield higher than 1 % after 24 h at 70 °C. Consequently, MgO/KOH was selected for further analysis following the experimental design described in the Experimental Section. The XRD pattern of the MgO/KOH is shown in Figure 1. The diffractogram represents a typical MgO crystalline solid (peaks at 37°, 43°, 62°, 75°, and 79°), which was stable for several days after synthesis. Despite previous studies showing a peak at around 38° representing K_2_CO_3_,^[14]^ this was not clearly observed in our results. A catalyst reuse study (described later) indicated that the catalysts were stable during the reaction, with no changes observed in the XRD pattern after use.

The results of the glycerolysis reaction using MgO/KOH are shown in Table 1. A central composite design (CCD) was performed to optimize the MAGs yield. CCD is an extension of a factorial design that enables fitting a second‐degree polynomial equation to the data. It offers several advantages compared to a full factorial two‐level design of experiments (DoE), including the ability to estimate the curvature of the response surface and more efficient optimization (requiring fewer runs). The variables were tested at five different levels: −α (minimum), −1 (low), 0 (center), +1 (high), and +α (maximum), with the coded actual values shown in the Experimental Section. The CCD is made rotatable by the choice of α so the variance of predicted response surface design is constant on spheres and the second‐order model can provide good predictions,^[36]^ which in this study was α=1.68.

**Table 1:** Design of experiments for glycerolysis reaction catalyzed by MgO/KOH.

Run	Temperature	Catalyst	Glycerol : oil	MAGs yield/%
1	−1	−1	−1	2.25
2	−1	−1	1	22.03
3	−1	1	−1	4.21
4	−1	1	1	16.14
5	1	−1	−1	12.07
6	1	−1	1	36.08
7	1	1	−1	16.14
8	1	1	1	38.97
9	−1.68	0	0	12.25
10	1.68	0	0	36.21
11	0	−1.68	0	22.05
12	0	1.68	0	34.06
13	0	0	−1.68	1.95
14	0	0	1.68	43.24
15	0	0	0	27.12
16	0	0	0	32.05
17	0	0	0	33.29

**Figure 2:**
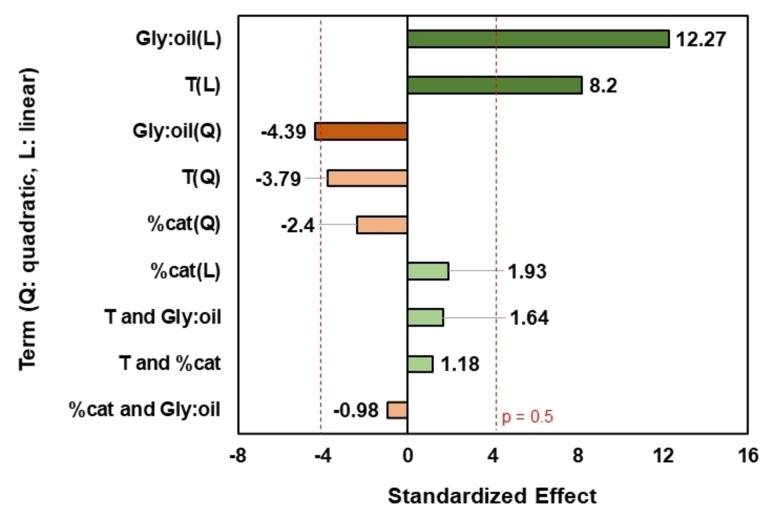
Pareto chart at low temperature (36–104 °C) and atmospheric pressure using MgO/KOH as catalyst. Dashed line represents the limit to significant values according to a confidence interval of 95 %.

The Pareto‐chart obtained (Figure 2) showed three significant terms at the considered confidence level (95 %), the glycerol:oil molar ratio terms (linear and quadratic) and temperature (linear). Consequently, two factors from the DOE had significant effect on reaction yield, the glycerol:oil molar ratio and temperature. To evaluate whether the model has any significant quadratic components, we need to test for curvature. As the curvature was significant, the yield is better described by a quadratic model than a linear model.

High temperature and excess glycerol are known to be key parameters in glycerolysis reaction, especially for heterogeneous catalysts. Studies have demonstrated the inefficiency of using these catalysts at low temperatures, which was shown by comparing glycerolysis and transesterification reactions. For glycerolysis, CaO, MgO, and Ca(OH)_2_ showed a poor performance below 100 °C, which can be attributed to these catalysts inability to extract hydrogen from glycerol at these temperatures.^[21]^ This hypothesis was given weight from the previous observation that the KOH catalyzed glycerolysis of soybean oil reached reaction equilibrium after 24 h at 80 °C,^[14]^ while the transesterification reaction using the same catalyst was efficient at 60–65 °C.[^37^, ^38^] These results may be a consequence of the catalysts being able to extract hydrogen from methanol at lower temperatures more easily than from glycerol.^[14]^ Additionally, excess glycerol would be essential to shift the equilibrium towards MAG formation as glycerolysis is a reversible reaction.^[14]^

In terms of selectivity, calculated as a percentage of the moles of desired product (MAG) divided by the moles of total products (MAG and DAG), results ranged from 30 to 50%. As expected, both selectivity and the yield of MAGs increased with the concentration of glycerol, with the highest selectivity observed at the highest glycerol:oil ratio tested. While previous studies utilising MgO/KOH as a catalyst have not published selectivity results, a study on glycerolysis of palm oil employing a comparable catalyst (metal oxide with low base loading: CuO‐nano+0.01 wt % NaOH) reached a similar selectivity of around 50 %.^[19]^

**Figure 3:**
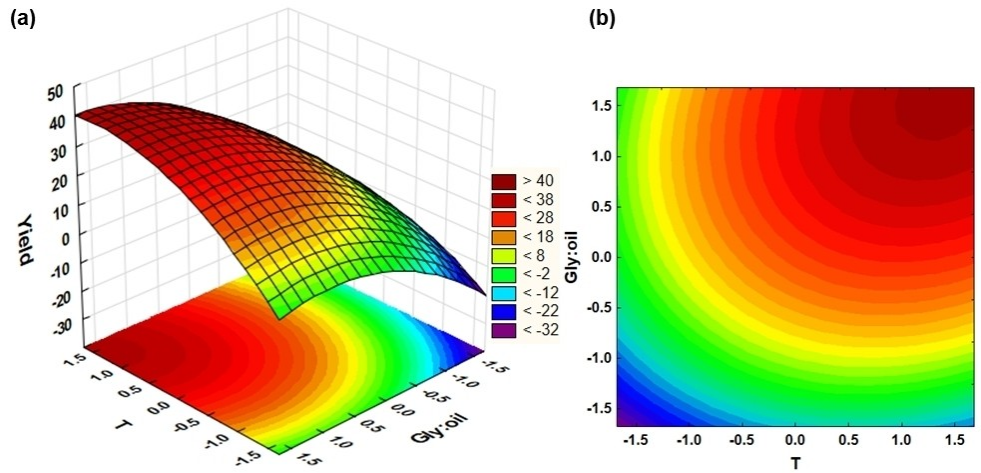
Optimization of monoglyceride yield by glycerolysis of rapeseed oil at low temperature (36–104 °C) and atmospheric pressure using MgO/KOH as catalyst and different glycerol:oil molar ratios (1 : 1–11 : 1): (a) response surface and (b) contour plot.

In addition to the individual effects of the three factors (temperature, glycerol:oil ratio, and catalyst amount) on reaction yield, their interaction as pairs were also evaluated. Response surface methodology was then applied to study interaction effects between the two independent variables with significant effects on the MAG yield. From Figure 3, it is possible to observe the maximum yield towards an increasing temperature and glycerol:oil ratio.

The position of maximum yield with precise conditions could not be displayed within the design limits (±1.68 levels). Initially, a full factorial DoE with three factors at two levels was conducted to estimate the main effects and interactions. Thus, runs 1–8 and 15–17 (Table 1) were evaluated. After observing a significant curvature, the additional experiments of the CCD were performed (runs 9–14), achieving a maximum surface area near the boundary of reaction conditions. MAGs yield was higher than 40% where temperature was between 90 and 100 °C and glycerol:oil ratio was close to 11. The negative values represent an error of the model due to some points being close to zero (Table 1).

Analysis of Variance (ANOVA) was performed on the 17 experimental runs to compare variances across the means (Table 2). To determine which terms could be excluded from the model aiming for the best fit, a backward elimination (from the lowest to the highest effect on MAGs yield) was conducted. The adjusted coefficient of determination (R^2^‐adjusted) was 0.86, which included the variables: glycerol:oil molar ratio (linear and quadratic), temperature (linear and quadratic), catalyst content (linear and quadratic), and interaction between temperature and glycerol:oil molar ratio. Consequently, although three out of nine terms showed significant effects according to Figure 2 using a confidence interval of 95% (p = 0.05), all but two of these nine variables were included in the fitting equation. The resulting quadratic model is shown in Eq. 1.

(1)

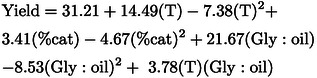




**Table 2:** ANOVA table obtained from glycerolysis design of experiments after backward elimination (R^2^=0.86).

Factor	SS^[a]^	DF^[a]^	MS^[a]^	F‐value	p‐value
T(L)	716.6	1	716.6	67.3	0.014
T(Q)	153.4	1	153.4	14.4	0.063
% cat(L)	39.5	1	39.5	3.7	0.194
% cat(Q)	61.5	1	61.5	5.8	0.138
Gly : oil(L)	1603.7	1	1603.7	150.6	0.006
Gly : oil(Q)	205.2	1	205.2	19.3	0.048
T and Gly : oil	28.6	1	28.6	2.7	0.243
Lack of fit	203.8	7	29.1	2.7	0.294
Pure error	21.3	2	10.6		
TotalSS	2895.0	16			

[a] Sum of squares. [b] Degrees of Freedom. [c] Mean Squares.

where ‘T’, ‘% cat’ and ‘Gly:oil’ correspond to the coded values of these variables.

F‐tests were conducted based on the degrees of freedom and mean squares or sum of squares in Table 2. The F distribution compares the ratio of two variances, i.e., how far the data are spread out from their mean. The first F‐test compared the ratio between the mean squares of the residue (lack of fit and pure error) and the regression (terms in Eq. 1) with the value obtained by the degrees of freedom using the F distribution table. The second F‐test compared the ratio between the sum of squares of the lack of fit and pure error with the value from F distribution table. As both tests were valid, the model shown in Eq. 1 is significant and could be used to predict MAG yield with a 95% level of confidence within the experimental limits studied. However, as the model predicts negative values around the lowest level for temperature and glycerol:oil molar ratio, which do not have a physical meaning, the response surface was used only to distinguish optimal values.

**Figure 4:**
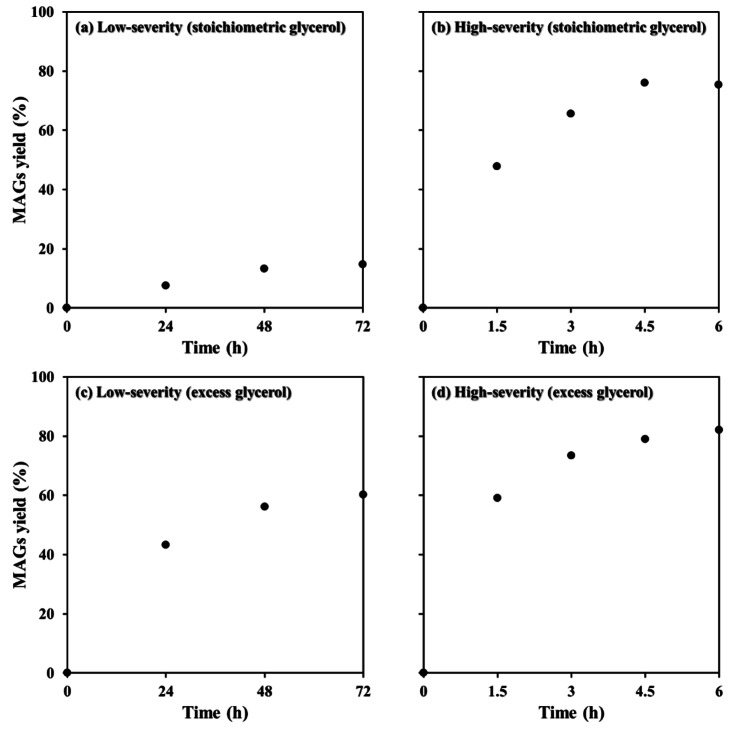
Glycerolysis reaction of rapeseed oil using MgO/KOH (3 %) as catalyst: (a) low‐severity with stoichiometric glycerol (2 : 1), (b) high‐severity with stoichiometric glycerol (2 : 1), (c) low‐severity with excess glycerol (11 : 1), and (d) high‐severity with excess glycerol (11 : 1).

For the following experiments, different reaction times and two reaction systems were evaluated. As the glycerol:oil molar ratio was the most significant variable, two levels were further studied varying the reaction time; stoichiometric glycerol (2 : 1) and excess glycerol (11 : 1). Figure 4a shows that glycerolysis reaction with stoichiometric glycerol concentration at low temperature and atmospheric pressure is extremely time‐consuming, achieving only 15 % yield after 72 h. However, a significantly higher reaction yield (76 %) was obtained after 4.5 h when performed in the autoclave (Figure 4b). When excess glycerol was used, glycerolysis in a round‐bottom flask (low temperature) achieved 60.1 % (Figure 4c), compared to 82.1 % (Figure 4d) in an autoclave reactor (high temperature and pressure). Consequently, the improvements in using excess glycerol were more evidenced in reactions performed at low temperature and pressure, but less significant at higher temperature and pressure when viscosity of the reaction medium is lower. Another significant difference was observed using the autoclave reactor, which reached MAG selectivity closer to 1, up to 0.95.

**Figure 5:**
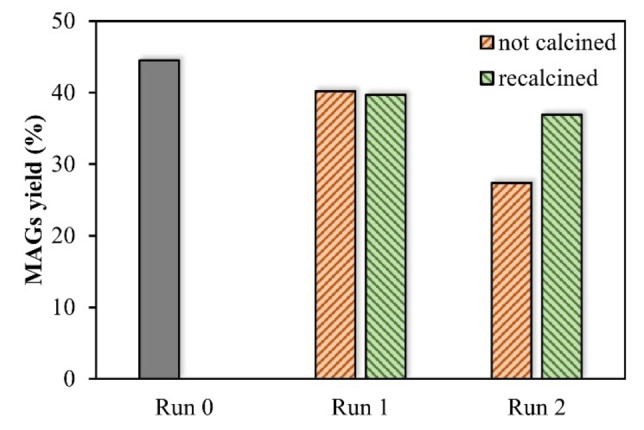
MgO/KOH catalyst reuse in glycerolysis of rapeseed oil.

Finally, a brief study on catalyst reuse was conducted using the low‐severity conditions with excess glycerol. The impact on the MAGs yield over three consecutive reactions is shown in Figure 5. Initially, the catalyst reuse was carried out by recovering the catalyst following the procedure described in the Experimental Section and drying the solid overnight. A slight decrease in MAGs yield was observed from the first reaction with fresh catalyst (run 0) to the first reuse attempt (run 1), although a greater reduction was evidenced in run 2. As the yield dropped after the second use (run 2) a new reuse procedure was evaluated where the used catalyst was recalcined prior to testing. This methodology showed a similar reaction yield between the first and second reuse reaction yield, which was 35% higher than without the calcination step. Although, even with a calcination step, the yield on reuse still decreased compared to the initial run, further refinement of the recalcination step may increase the reusability of MgO/KOH. Comparing both methods of catalyst reuse, the reduction of MAGs yield could be partially explained by the reduction in surface area. From an initial 40.1 m^2^/g, the surface area dropped to 32.6 m^2^/g when the catalyst was only washed, while it reached 38.1 m^2^/g when it was recalcined. Moreover, in pursuit of a sustainable catalyst reuse method, previous studies have demonstrated the possibility of recovering the KOH solution^[39]^ from the synthesis process to obtain fresh catalysts, thereby potentially reducing costs further.

A catalyst leaching study was also conducted, although this was hampered by the presence of Mg and K in the rapeseed oil. The investigation showed that the Mg concentration dropped from 6 ppm in the rapeseed oil prior to use to less than 1 ppm after reactions, whereas K concentrations were found to be 235 ppm in the commercial oil, 204 ppm after the first use and 215 ppm after the first reuse experiment. Considering the initial metal concentrations were higher than those found after the reaction, no significant variation was observed, indicating that Mg and K do not leach into the reaction products during the reaction for low severity glycerolysis with excess glycerol (11 : 1) at 90 °C.

### Validation of Microalgal Oil

Having explored the reaction variables using rapeseed oil, microalgal oil reaction was then studied using optimum values for low severity glycerolysis; excess glycerol (11 : 1) and 90 °C. The low level (not minimum) of catalyst was chosen (3 %) although this variable was not significant within the design matrix limits, because further mass transfer limitations were expected for microalgae as the lipids were not completely purified into an acylglycerol mixture. For comparison, the reaction using excess glycerol (11 : 1) was also studied in the autoclave reactor.

**Figure 6:**
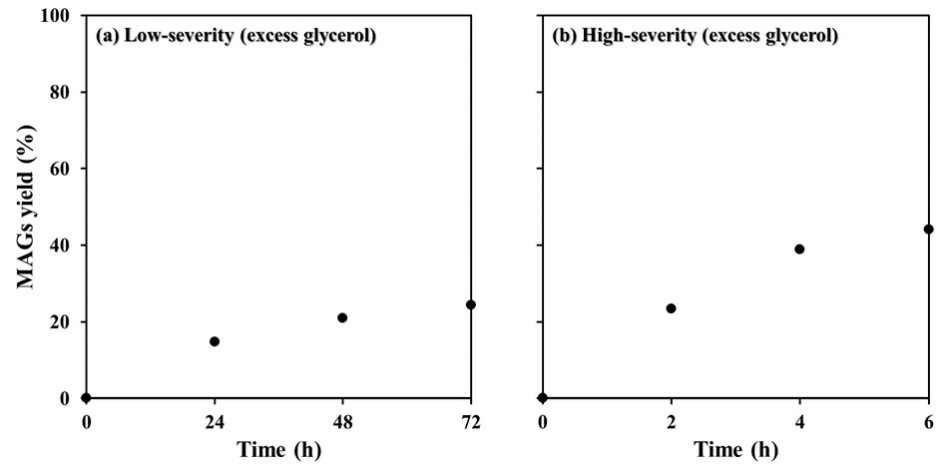
Glycerolysis reaction of microalgal oil using MgO/KOH (3 %) as catalyst: (a) low‐severity with excess glycerol (2 : 1), and (b) high‐severity with excess glycerol (11 : 1).

Low‐severity reactions under atmospheric pressure at 90 °C were found to give a MAG yield of 24.3 % (Figure 6a) after 72 h. High‐severity reactions at an initial 20 bar using the autoclave reactor achieved 44.1 % (Figure 6b). Both reaction systems achieved around half the value obtained for the glycerolysis of rapeseed oil under the same reaction conditions. By comparing a partially purified microalgal oil to a commercial vegetable oil, differences in the fatty acid profile and the amount of free fatty acids or other compounds may influence oil solubility. Also, *Scenedesmus* sp. reaction curves show a higher inclination (higher angle from x‐axis) between the last two points of the graphs in Figure 5, which may indicate that these reactions are further from the equilibrium conditions. Consequently, the reaction yield could be expected to increase at longer reaction times. As expected, the MAG yield was higher in the autoclave reactor, which also had the advantage of an inert atmosphere, which avoids the oxidation of PUFAs‐rich oil during processing.

Comparing results in the two reaction systems, the reaction in the round‐bottom flask took 72 h to reach a MAG yield similar to the reaction in the autoclave after 2 h (23.4 %). The 36‐fold longer reaction time to reach this value demonstrates how higher temperature, pressure, and intense agitation increase the reaction rate. Nonetheless, reaction yields obtained for microalgal oil were comparable to heterogeneous‐catalyzed glycerolysis of vegetable and seed oils, that have reported a MAG yield of around 40 %.^[1]^ Considering that the results presented here were achieved for microalgal oil obtained by a simple extraction and purification method (see Experimental Section), a similar yield to commercial oils is a promising outcome.

As mentioned previously, both homogeneous and heterogeneous glycerolysis require high temperatures for efficient MAG and DAG synthesis, which is a challenge for product stability. Liu et al.^[40]^ performed NaOH‐catalyzed glycerolysis in waste cooking oil at low temperature (55 °C) using acetone as solvent and observed that MAGs were the major product. Although products showed promising physicochemical and emulsifying properties, further research is required to evaluate low temperature base‐catalyzed glycerolysis. On the other hand, enzymes can reach high conversion at low temperatures but are expensive. Da Silva et al.^[41]^ compared glycerolysis of buriti oil catalyzed by MgO and an immobilized lipase, showing that the enzymatic route reached a higher conversion and selectivity towards MAGs. While the MAG yield was 87.2 % for enzymatic glycerolysis at 55 °C, the maximum yield using MgO was 57.3 % at 210 °C, both after 8 h. Also, despite applying a low‐pressure N_2_ flow to provide an inert atmosphere inside the 50 mL two‐necked round bottom flask, alkaline glycerolysis showed significant carotenoid degradation. Consequently, these catalytic routes have advantages and disadvantages to consider in an economic evaluation coupled with product quality.

To assess product stability, fatty acid profiles were obtained, as shown in Table 3. Despite having a different fatty acid profile, both rapeseed and microalgal oil have a majority of unsaturated fatty acids (MUFAs and PUFAs). Reactions at low temperature and atmospheric pressure showed degradation of PUFAs and a consequent increase in monounsaturated fatty acids and saturated fatty acids content. As *Scenedesmus* sp. lipids have around 2/3 of unsaturated fatty acids, reactions in the presence of oxygen are not suitable for higher‐value applications. If a lower temperature was employed, the time required to achieve significant MAG yield would substantially increase. Hence, the best conditions for microalgal oil processing would be reactions under an inert atmosphere. Unlike the transesterification reaction, glycerolysis has been reported to be efficient with high free fatty acid content (5–60 %),^[42]^ so a prior neutralization step is not required. Additionally, improvements in purification could increase the acylglycerol content of extracted and purified lipids. The most unstable fatty acids were found to be those with 16 carbons (Table 3). For the lower temperature reaction in air, a PUFA decrease from 50.2 to 36.6 % was observed while palmitic acid (C16:0) increased from 26.1 to 36.6%. In the autoclave, this instability was not detected due to the inert atmosphere preventing oxidation. Consequently, further research is required to establish a cost‐efficient glycerolysis reaction of microalgae containing high amounts of these essential fatty acids (omega 3, ‐6, and ‐9) at low temperature. However, reaction in an autoclave with inert atmosphere was able to achieve significant MAGs yield avoiding fatty acid oxidation. Among the various reaction routes and synthesis strategies for producing MAGs and DAGs from lipids, including alcoholysis, esterification, and glycerolysis, the latter stands out due to its potential for utilizing both a more sustainable catalyst (heterogeneous) and a biorenewable reaction byproduct (glycerol).^[1]^

**Table 3:** Profiles and total saturated (SFA), monounsaturated (MUFA), and polyunsaturated (PUFA) fatty acids of rapeseed oil and microalgal oil compared to the mixture of acylglycerols after the glycerolysis reaction using microalgal oil.

Fatty acids		Rapeseed oil	*Scenedesmus* sp. lipids	Low T, P_atm_			High T, P_initial_=20 bar		
				24 h	48 h	72 h	2 h	4 h	6 h
C15 : 0	Pentadecanoic acid	–	4.1	6.7	7.4	5.7	4.8	5.4	3.2
C16 : 0	Palmitic acid	3.9	26.1	30.1	32.5	36.6	24.8	25.0	24.9
C16 : 1c	Palmitoleic acid	0.8	0.9	1.4	2.2	2.2	–	–	0.4
C16 : 2‐C16 : 4	Polyenoic fatty acids	2.4	7.0	11.2	8.7	1.4	6.4	10.9	7.4
C18 : 1c	Oleic acid	57.1	16.4	18.3	17.4	18.4	17.2	16.4	17.3
C18 : 2c	Linoleic acid	22.3	20.5	14.2	16.3	14.4	21.8	19.0	21.1
C18 : 3γ	Gama‐linolenic acid	1.5	1.0	–	0.4	0.5	1.1	1.0	1.0
C18 : 3α	Alpha‐linolenic acid	10.6	21.2	15.0	13.6	13.3	21.6	17.4	21.5
C20 : 0	Arachidic acid	0.2	1.0	0.1	0.2	–	1.3	1.4	1.2
C20 : 1	Eicosenoic acid	0.9	0.6	1.6	0.6	0.5	1.0	1.6	1.4
C20 : 3	Eicosatrienoic acid	0.1	0.5	1.4	0.7	0.3	0.6	1.8	0.6
C22 : 0	Behenic acid	0.2	0.7	–	–	–	0.5	0.2	0.4
SFA		4.3	31.9	36.9	40.1	42.3	31.3	32.0	29.7
MUFA		58.8	17.9	21.3	20.2	21.1	18.2	18.0	19.1
PUFA		36.9	50.2	41.8	39.7	36.6	51.5	50.0	51.2

### Conclusions

This study has demonstrated for the first time that heterogeneous catalysis is a viable alternative to enzyme catalysis for the production of high value products from microalgae oils. The MgO/KOH catalyst was found to give promising yields for the glycerolysis of microalgae oils under the studied conditions. At low temperature and pressure, excess glycerol was found to be the most significant variable, with higher temperatures increasing MAG production. Higher temperature and pressure were found to significantly improve the reaction yield, even at stoichiometric glycerol : oil ratios; with an inert atmosphere essential to prevent degradation of the products. Glycerolysis of microalgal oil in an autoclave reactor using MgO/KOH as catalyst reached 44 % MAGs yield. In heterogeneous catalysis, the reuse of catalyst is a major goal to reduce both environmental and economic impacts, hence preliminary results of this catalyst reuse are shown in this study. A simple recovery step has proven insufficient to maintain reaction yield, although a recalcination step showed promising results.

This proof‐of‐concept study highlights the importance of heterogeneous catalysis in biomass valorization and has the potential to open up a new field studying microalgae oil feedstocks to produce high value products. The advantage of using inexpensive, robust, scalable, solid catalysts provides a more economical process compared to enzymatic catalysts that can be expensive and difficult to scale up. An economic evaluation of large‐scale production using different systems would provide insights into the viability of this process. The large body of previous research on heterogeneous catalysts for biodiesel production from vegetable oils can contribute to the growth and development of this research field in the future.

## Experimental Section

### Rapeseed Oil

#### Catalysts Preparation for Glycerolysis Reaction

**Basic metal oxide : MgO/KOH** Mg(OH)_2_ was calcined in a tubular furnace under static air for 2 h, at 600 °C (heating ramp 10 °C min^−1^). The obtained MgO (10 g) was rehydrated by refluxing in distilled water (125 mL) for 3 h, dried (90 °C, 24 h), and calcined again under the same conditions.^[43]^ Suspension of 10 g MgO in 40 mL of methanol was followed the addition of 5 mL aqueous KOH solution (0.2 g/mL). The slurry was stirred at 600 rpm for 3 h at 40 °C, then heated to 120 °C for 3 h. The obtained solid was stored in a desiccator and was activated by calcination in static air at 650 °C for 8 h before use.^[14]^

**Acid metal oxide : Nb_2_O_5_/H_3_PO_4_** Nb_2_O_5_.H_2_O (3 g) was added to a 10 mL H_3_PO_4_solution (1 mol L^−1^) and stirred for 48 h at ambient temperature. The mixture was then dried in an oven for 72 h at 90 °C.^[44]^

### Catalyst Characterization

#### Surface Area Measurements

Surface area analysis was carried out using a Quantachrome Chem BET chemisorption analyzer equipped with a thermal conductivity detector (TCD). Samples (50 mg) were degassed at 150 °C for 3 h before analysis using a Quantachrome FLOVAC Degasser. The amount of N_2_ emitted was assumed to amount to half a monolayer coverage.

#### X‐Ray Powder Diffraction

X‐ray powder diffraction measurements were performed using a PANalytical X′pert Pro diffractometer with Ni‐filtered Cu Kα radiation source operating at 40 kV and 40 mA. Patterns were recorded over the range of 10–80° 2θ using a step size of 0.0168°. All patterns were identified using the ICDD database.

#### Glycerolysis Reaction

**Low‐severity reaction** Screening of different catalysts for monoglyceride synthesis from rapeseed oil (2 mL, Sainsbury′s) was conducted using glycerol (0.39 g), acetone (8 mL), and 5 wt % metal oxide catalyst (0.09 g). The reaction was performed in a two‐neck round bottom flask (100 mL) equipped with a vertical condenser and immersed in a water bath. Reaction conditions were: 70 °C, 24 h, under vigorous magnetic stirring. Following the reaction, acetone was evaporated, and 5 mL of a hexane/isopropanol mixture (15 : 1, v : v) was added.^[14]^ The catalysts were recovered using a microfilter (0.45 μm). In catalyst reuse experiments, the catalyst was recovered through filtration followed by washing with warm hexane/isopropanol mixture (heated to 70 °C) and ethyl acetate^[45]^ to remove any remaining acylglycerols. To investigate catalyst leaching, Mg and K concentrations in the reaction mixture at the end of the reaction were determined using Inductively Coupled Plasma – Mass Spectrometry (ICP‐MS).

A design of experiments study was conducted using the best performing MgO/KOH catalyst to evaluate the effect of reaction parameters (glycerol : oil ratio, temperature, and catalyst loading), with triplicates in the center point (Table 4). Solvent (acetone) volume was fixed. A CCD was used, resulting in 14 different experimental conditions. As this design contains twice as many star points as there are factors in the design, it leads to 2^3^+2×3=14 experiments. Summing up the center points, a total of 17 experiments were conducted. The results were analyzed using Statistica 7 from Statsoft® with 95 % confidence interval. Finally, reaction time was evaluated using optimized experimental conditions. After determining when the reaction reaches equilibrium, this condition was repeated for validation. Thus, duplicates of experiments at optimum conditions were performed to validate MAGs yield.

**Table 4:** Levels of the independent variables in the Design of Experiments (α=1.68).

			Coded actual values				
Independent variables factor	Unit	Code	Min. (−α)	Low level (−1)	Medium level (0)	High value (+1)	Max. (+α)
Glycerol : oilmolar ratio	–	Gly : oil	1	3	6	9	11
Catalyst content	wt %^[a]^	%cat	2.3	3	4	5	5.7
Temperature	°C	T	36	50	70	90	104

[a] Sum of squares.

**High‐severity reaction** Monoglycerides synthesis from rapeseed oil (6 mL) and glycerol (stoichiometric −1.17 g and excess −6.42 g) with MgO/KOH catalyst (0.16 g) and acetone (24 mL) was performed in a high‐pressure stainless‐steel autoclave with a 50mL glass liner (Parr). The reactor was sealed, purged 3x with N_2_ at 20 bar, heated to 200 °C, and kept for 1.5–6 h at 500 rpm agitation. Before the reactor was depressurized, the system was cooled to room temperature in an ice water bath. The product was then separated following the procedure previously described for the low‐severity reaction.

**Product analysis** MAGs, DAGs, and TAGs were quantified using high‐performance liquid chromatography (HPLC) using an Agilent 1260 Infinity chromatograph equipped with a 250 mm Agilent HC‐C18 column with a 4.6 mm inner diameter and an octadecylsilane phase and a diode‐array UV detector (DAD). Two mobile phases with gradient profile were applied: (A) methanol, from 100 to 0 % elution in 60 min, and (B) i‐propanol/n‐hexane (5 : 4 v/v), from 0 to 100 % elution in 60 min, followed by isocratic elution for a further 10 min. The samples were injected (10 μL) at room temperature after having been diluted to 3 % (p/v) in i‐propanol/n‐hexane (5 : 4 v/v).^[46]^ Calibration was performed using methyl oleate, monoolein, diolein, and triolein standards at five different concentrations.

#### Microalgal Oil

The optimized reaction conditions from the vegetable oil experiments were used to investigate the production of MAGs from *Scenedesmus* sp. LF01 microalga, grown and donated as wet biomass by Algae Biotecnologia Ltd Microalgae inoculum was provided by the Federal University of São Carlos (UFSCar). Wet biomass was dried using aspray dryer (DR‐0,4AIRSpray Process). Lipid extraction was then conducted using hexane (60 °C, 1 h) assisted by ultrasound (Unique USC‐2800 40 kHz). The residual biomass was filtered, andhexane was evaporated in a rotary evaporator (IKA RV 10) leaving behind the extracted lipids. Before reaction, extracts were purified using a chromatography column packed with silica gel and bentonite as the stationary phase, and chloroform as mobile phase.^[47]^

After evaluation of catalytic performance using rapeseed oil, the most promising conditions identified were assessed for the production of MAGs from *Scenedesmus* sp. oil. Products on both low and high‐severity reactions were quantified according to ASTM D6584^[48]^ and EN14105^[49]^ using a gas chromatograph fitted with a flame ionization detector (Agilent GC‐FID 7890A). Fatty acid profiles were also obtained using a GC‐FID (Agilent GC‐FID 6850), according to the procedure described previously.^[50]^

## Conflict of Interests

The authors declare no conflict of interest.

1

## Data Availability

The datasets generated during the current study are available free of charge through the Cardiff University Research Portal https://doi.org/10.17035/d.2024.0215513966.
